# Hyperchaotic Image Encryption Based on Multiple Bit Permutation and Diffusion

**DOI:** 10.3390/e23050510

**Published:** 2021-04-23

**Authors:** Taiyong Li, Duzhong Zhang

**Affiliations:** School of Economic Information Engineering, Southwestern University of Finance and Economics, Chengdu 611130, China; zhangduzhong@swufe.edu.cn

**Keywords:** hyperchaotic, image encryption, permutation, diffusion, multiple bit operation

## Abstract

Image security is a hot topic in the era of Internet and big data. Hyperchaotic image encryption, which can effectively prevent unauthorized users from accessing image content, has become more and more popular in the community of image security. In general, such approaches conduct encryption on pixel-level, bit-level, DNA-level data or their combinations, lacking diversity of processed data levels and limiting security. This paper proposes a novel hyperchaotic image encryption scheme via multiple bit permutation and diffusion, namely MBPD, to cope with this issue. Specifically, a four-dimensional hyperchaotic system with three positive Lyapunov exponents is firstly proposed. Second, a hyperchaotic sequence is generated from the proposed hyperchaotic system for consequent encryption operations. Third, multiple bit permutation and diffusion (permutation and/or diffusion can be conducted with 1–8 or more bits) determined by the hyperchaotic sequence is designed. Finally, the proposed MBPD is applied to image encryption. We conduct extensive experiments on a couple of public test images to validate the proposed MBPD. The results verify that the MBPD can effectively resist different types of attacks and has better performance than the compared popular encryption methods.

## 1. Introduction

In the current era of Internet and big data, billions of images are produced, stored and transmitted every day. How to protect image content from illegal acquisition, especially for military, medical, and privacy purposes, has become a hot topic in recent years. Because of some attributes of images, such as high redundancy, strong correlation, and bulky data, traditional encryption methods for common text and data are usually not the best choice for image encryption. In recent years, various chaos-based image encryption approaches have emerged and they have been demonstrated very effective in improving image security. The reason why chaotic image encryption has become so popular is that chaotic systems have some characteristics that are very suitable for image encryption, such as extreme sensitivity to initial values, unpredictability, pseudorandomness, and ergodicity [1,2,3].

In chaotic image encryption, chaotic sequences are generated from the chaotic systems and they usually are applied to change the positions and/or values of image data. Early schemes usually used single low-dimensional chaotic systems, such as Logistic map, Tent map, Baker map, Cat map, etc., to encrypt images [4,5,6,7,8]. For example, Chen et al. extended 2D Cat map to a 3D one and designed a fast symmetric encryption approach, and the experiments demonstrated the approach was superior to the compared methods in terms of security and speed [4]. Pisarchik et al. proposed a pixel-by-pixel image encryption with Logistic maps [7]. Although these schemes achieved satisfactory encryption results at that time, the relatively simple structure of low-dimensional chaotic systems made them have a certain risk of being cracked. To solve this issue, possible directions are to use a more complex chaotic system or to combine two or more simple chaotic systems. In recent years, a variety of researchers have attempted to improve the performance of image encryption in these two directions. According to the theory of chaos, Lyapunov exponent (LE) can be used to characterize the variable of a chaotic/hyperchaotic system. A dynamic system is chaotic if it has one positive LE, while it is hyperchaotic if it has two or more positive LEs. In general, image encryption schemes based on hyperchaotic systems are more secure than those with chaotic systems. In real-world encryption, Lorenz system and its extensions are among the most popular chaotic/hyperchaotic systems [9,10,11,12]. Wang and Zhang applied a 4D Lorenz-like hyperchaotic system with two positive LEs and genetic recombination to image encryption [10]. Li et al. used 5D and 7D hyperchaotic systems, dynamic filtering, DNA permutation and bit cuboid operations for image encryption, and the experimental results prove the effectiveness [13,14]. Unlike most schemes that carry out encryption in spatial domain, Wu et al. used 2D discrete wavelet transform (2D DWT) and a 6D hyperchaotic system to encrypt images in both spatial domain and frequency domain [15]. In addition to integer-order hyperchaotic systems, fractional-order hyperchaotic systems are becoming more and more popular with image encryption [16,17,18,19]. Zhu and Sun proposed a Logistic-Tent map for image encryption [20]. Luo et al. cooperated a piecewise linear chaotic map and a 4D hyperchaotic map for parallel image encryption [21]. Other combinations include Henon-Sine map [22], Logistic map and Lu system [23], Logistic-Sine map [24,25,26,27,28,29], Logistic-Tent-Sine map [30,31], Rossler-Sine map [32], etc. These combinations have been proven effective in improving the security of encryption.

The aim of image encryption is to prevent unauthorized users from discovering any meaningful content in the image. In other words, encrypted images are entirely random-like for them. There are many operations to convert informative images (plain images) to random-like ones (cipher images), among which permutation and diffusion are two major ones. Permutation changes the positions of image content, while diffusion changes the values of images. Most existing image encryption schemes adopted both the operations, separately or jointly, to achieve good security [33,34,35,36,37]. Among them, pixel-level (8 bits) data or bit-level (1 bit) data are the most widely used encryption units. In recent years, DNA computing has been introduced into image encryption; hence, DNA-level (2 bits) data has also be used to encrypt images [38,39,40,41,42]. Most studies focus on one or two bit levels in image encryption and the bit levels of encrypted data need to be enhanced to improve the effectiveness of image encryption. In fact, besides the mentioned 1 bit, 2 bit and/or 8 bit encryption operations, other multiple bit data, such as 3–7 bit data, can also be used to permutate and/or diffuse images. More bit-level data enhances the diversity of encrypted units and may have the potential to improve encryption performance. However, few existing studies have paid attention to this point.

Motivated by the above analysis, this paper proposes multiple bit permutation and diffusion, namely MBPD, for hyperchaotic image encryption. We first extend a modified 3D Lorenz chaotic system to a 4D hyperchaotic system with 3 positive LEs, and its characteristics are analyzed. Second, the hyperchaotic system is used to generate a hyperchaotic sequence for the consequent encryption operations. The initial values of the hyperchaotic system are considered as keys for the purpose of encryption. Then, the operations of multiple bit permutation and multiple bit diffusion are presented to encrypt images. The MBPD treats several bits (e.g., 3 bits) as a processing unit for permutation and diffusion, and different lengths of bits can be chosen for encryption. For the permutation, the order of the hyperchaotic sequence is used to scramble the multiple bit data, while the sequence will be converted into an integer mask for the diffusion. Finally, the proposed MBPD with different lengths of bits is applied to image encryption to improve security.

The contributions of this paper are the following:

(1) A new 4D hyperchaotic system with 3 positive LEs is presented, and some related hyperchaotic characteristics are analyzed.
(2) Multiple bit permutation and diffusion is proposed for image encryption, which is very different from most existing image encryption schemes that encrypt images only with 1 bit, 2 bit and/or 8 bit data. To the best of our knowledge, it is the first time that multiple bit operations are proposed for image encryption.
(3) Extensive experiments demonstrate that the proposed MBPD significantly outperforms the state-of-the-art compared image encryption schemes in terms of the evaluation indicators.

The rest of this paper is organized as follows: Section 2 presents a new 4D hyperchaotic system with 3 positive LEs. Section 3 proposes MBPD and details the encryption steps. In Section 4, experimental results are reported and analyzed. Finally, we conclude the paper in Section 5.

## 2. Presented 4D Hyperchaotic System

### 2.1. Lorenz System

Since the chaotic attractor was first found by Lorenz in 1963, chaos theory has attracted researchers from many fields, such as economics, mathematics, physics, and communications [9]. The initial Lorenz system has been extended to many versions. One modified generalized Lorenz system is formulated as Equation (1) [43].

(1) $$\left\{\begin{array}{c} \dot{x}=-ax+by\\ \dot{y}=cx+dy-xz\\ \dot{z}=-ez+x^{2} \end{array},\right.$$

where *a*, *b*, and *e* are positive real constants, and *c* and *d* are real parameters meeting $d>-\frac{bc}{a}$ [44]. By introducing a 1D linear system to Equation (1), a new 4D system can be obtained, as Equation (2).

(2) $$\left\{\begin{array}{c} \dot{x}=-ax+ay\\ \dot{y}=bx+cy-xz\\ \dot{z}=-dz+x^{2}\\ \dot{w}=ey+fw \end{array}.\right.$$

In this system, *a*, *b*, *c*, *d*, and *f* are real constant parameters, while *f* is a coupling parameter. When the parameters (a,b,c,d,e,f)=(35,7,35,5,1.5,1), the system has the following LEs: $LE_{1}=1.284559$, $LE_{2}=0.937533$, $LE_{3}=0.007986$, and $LE_{4}=-38.230078$. Since three LEs are positive, the system is hyperchaotic [44].

### 2.2. 4D Hyperchaotic System

Although Equation (2) is hyperchaotic, the introduced component *w* will increase exponentially after a certain number of iterations, and then its value will become positive infinity and its applications will be limited. To cope with this issue, we modify the fourth item of Equation (2) and add the component *w* to the first equation. A new 4D system can be obtained, as shown in Equation (3).

(3) $$\left\{\begin{array}{c} \dot{x}=-ax+ay+w\\ \dot{y}=bx+cy-xz\\ \dot{z}=-dz+x^{2}\\ \dot{w}=ey+fwsin\left(w\right) \end{array},\right.$$

where parameters a−f are the same as Equation (2).

**Figure 1:**
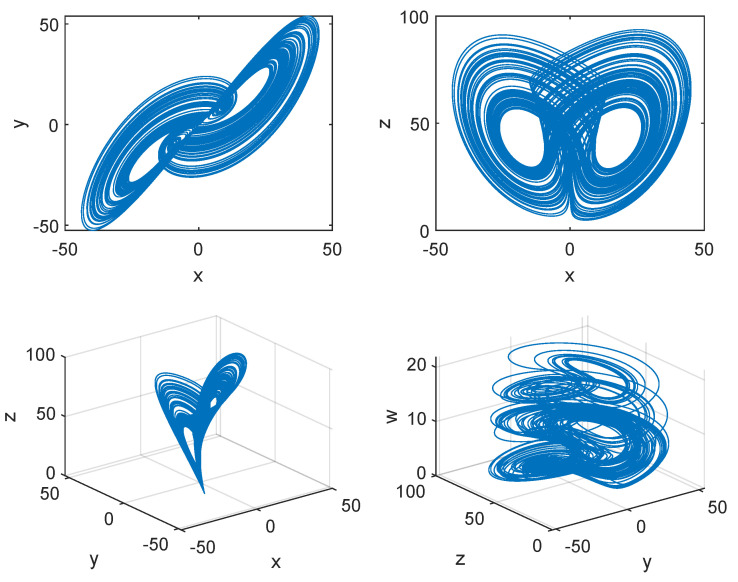
Attractors of the presented 4D hyperchaotic system with the parameters (a,b,c,d,e,f)=(35,7,35,5,1.5,1) and initial values $\left(x_{0},y_{0},z_{0},w_{0}\right)=\left(0.12,0.23,0.34,0.45\right)$.

We use the 4th-order Runge-Kutta method to plot the attractors of the presented 4D hyperchaotic system with parameters (a,b,c,d,e,f)=(35,7,35,5,1.5,1) and initial values $\left(x_{0},y_{0},z_{0},w_{0}\right)=\left(0.12,0.23,0.34,0.45\right)$ in 2D space and 3D space, as shown in Figure 1. From this figure, we can see that the component *w* falls within an appropriate range.

**Figure 2:**
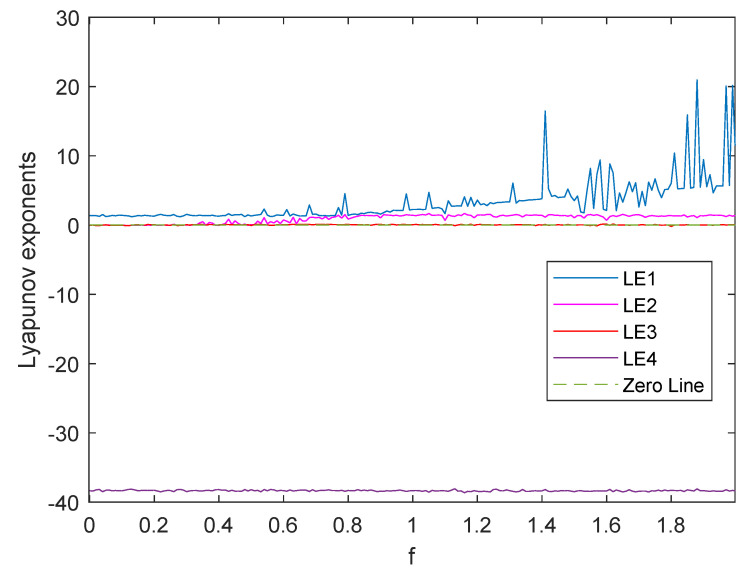
Dynamics of Lyapunov exponents of the proposed 4D hyperchaotic system with the parameters (a,b,c,d,e)=(35,7,35,5,1.5), variable *f* from 0 to 2, and initial values $\left(x_{0},y_{0},z_{0},w_{0}\right)=\left(0.12,0.23,0.34,0.45\right)$.

By using Wolf’s method [45], we fix (a,b,c,d,e)=(35,7,35,5,1.5) and let *f* vary from 0 to 2 to plot the dynamics of LEs, as shown in Figure 2. We can see that the new system has three positive LEs in many ranges. For example, when f=1, the LEs of the system are $LE_{1}=2.253019$, $LE_{2}=1.406374$, $LE_{3}=0.054342$, and $LE_{4}=-38.339706$ and the three positive LEs ($LE_{1}$, $LE_{2}$, and $LE_{3}$) are much larger than the corresponding positive LEs of Reference [43]. Therefore, the new system is also hyperchaotic, and it is better than Equation (2).

In this paper, we will use the new 4D hyperchaotic system for image encryption. The reasons lie in: (1) Although it has only 4 dimensions in total, it has 3 positive LEs. The hyperchaotic characteristics make it very suitable for image encryption. (2) It has a simpler mathematical form when compared with some hyperchaotic systems of higher dimensions. (3) All the components fall within appropriate ranges, making it easy to sort for permutation and convert hyperchaotic sequences into integers for diffusion.

## 3. MBPD: Multiple Bit Permutation and Diffusion

This section will detail the steps of multiple bit permutation and diffusion for image encryption, including how to generate the hyperchaotic sequence, operations of multiple bit permutation and multiple bit diffusion, and the encryption algorithm.

### 3.1. Hyperchaotic Sequence Generation

In chaotic image encryption, a chaotic sequence is required to generate index for permutation and a mask for diffusion. Given the parameters and the initial values, we use the Fourth-order Runge-Kutta method and an interval of 0.001 to solve the presented 4D hyperchaotic system in Section 2.2 and then construct the hyperchaotic sequence for encryption. The detailed steps are as follows:

Step 1: Given the initial values $IV=\{x_{0},y_{0},z_{0},w_{0}\}$, we solve the 4D hyperchaotic system to obtain long enough state values. The state values in the i−th iteration can be denoted as $s_{i}=\left\{x_{i},y_{i},z_{i},w_{i}\right\}$.
Step 2: To remove the adverse effects, the state values obtained by the first $it_{0}$ iterations are discarded.
Step 3: When the iteration terminates, we can get a hyperchaotic sequence *H* by concatenating all the $s_{j}\left(j=1,2,\cdots ,N\right)$ as Equation (4):
  (4) $$\begin{array}{cc} H=\{s_{1},s_{2},\cdots ,s_{N}\} & =\{x_{1},y_{1},z_{1},w_{1},\cdots ,x_{N},y_{N},z_{N},w_{N}\}\\ \; & =\{h_{1},h_{2},h_{3},h_{4},\cdots ,h_{4N-3},h_{4N-2},h_{4N-1},h_{4N}\}, \end{array}$$
  where *N* is the iteration times excluding $it_{0}$.
Step 4: Since the elements in *H* come from different equations in Equation (3) and, hence, have different ranges, we use the following formulation to further map each element in *H* to a uniform interval [0,1).
  (5) $$h_{i}=\left|h_{i}\times 10^{8}\right|-\left\lfloor \left|h_{i}\times 10^{8}\right|\right\rfloor ,$$
  where $\left|\cdot \right|$ and $\left\lfloor \cdot \right\rfloor$ are the mathematical computation of absolute value and flooring, respectively.

It can be seen that each element in *H* is a real value in [0,1). With an element $h_{i}$, we can use the following formula to map it to an integer *I* in the range of [0,N]:

(6) $$I=\lfloor \left(\left(\left|h_{i}\right|-\left\lfloor \left|h_{i}\right|\right\rfloor \right)\times 10^{14}\right)\rfloor \%N,$$

where % is the modulo operation.

### 3.2. Multiple Bit Permutation

Permutation is to rearrange the image content on a certain basis. For the permutation in chaotic image encryption, the positions of the image data to be permuted are usually determined by an index vector that can be obtained by sorting a hyperchaotic sequence. Typical permutation is conducted on pixel-level, DNA-level, and/or bit-level data [46]. The pixel-level data and DNA-level data in the current encryption technique actually refer to 8-bit data and 2-bit data, respectively. Few studies have focused on other numbers of bit data for encryption, such as 3–7 bits. In this paper, multiple bit permutation means conducting permutation on different numbers of bit data. *n*-bit permutation refers to using *n* bits as a minimum permutation unit.

All multiple bit operations require a bit stream of an image. Without loss of generality, given a bit stream *B* of length *L*, a hyperchaotic sequence *H*, and the number of bits to be permutated *n*, the first step is to calculate the number of permutation units PU and the remaining bits RB by PU=⌊L/n⌋ and RB=L%n, respectively. It is clear that RB<n. Then, the PU units need to rearrange according to the index of sorting PU values in *H* and the RB bits can be embedding into the rearranged bit stream at a position decided by a value in *H*.

The *n*-bit permutation can be described as Algorithm 1:
**Algorithm 1***n*-bit permutation.**Input:** 
a bit stream *B*, a hyperchaotic sequence *H*, and the number of bits to be permuted in a unit *n***Output:** 
a permutated bit stream PB, the number of used elements PU in *H*1:**function**BitPermute(*B*, *H*, *n*)2:    *L*← length(*B*); //length of *B*3:    PU←⌊L/n⌋;4:    RB←L%n;5:    PB←reshape(B(1:PU∗n),[PU,n]); //reshape the first PU∗n bits in *B* into a vector PB having PU*n*-bit units6:    [v,idx]←sort(H(1:PU)); // ascending sort to get the index vector idx7:    PB(1:idx)←PB; // permute the PU units8:    PB←reshape(PB,[1,PU×n]); // reshape the matrix PB to a bit stream9:    **if** RB<>0 **then**10:        PU←PU+1;11:        Generate a random position pos in the range of [1,PU] from H(PU) via Equation (6);12:        Insert the remaining RB bits B(L−RB+1:L) into PB at pos;13:    **end if**14:    **return** PB, PU;15:**end function**

When *n* equals 1 or 8, Algorithm 1 degenerates to bit-level permutation or pixel-level permutation. Hence, the common bit-level permutation and pixel-level permutation are the special cases of Algorithm 1.

**Figure 3:**
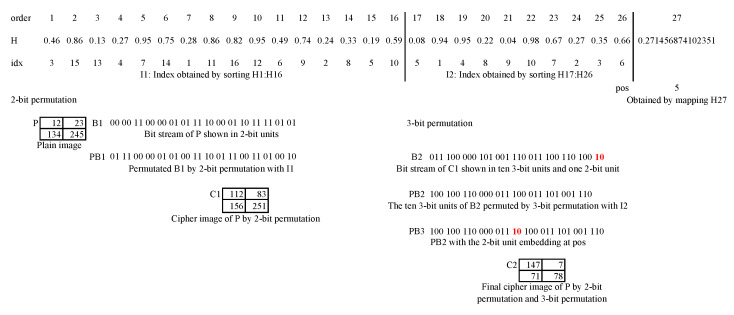
Illustration of 2-bit and 3-bit permutation.

Here, we take 2-bit permutation and 3-bit permutation as an example to illustrate the detailed permutation procedure, as shown in Figure 3.

A 2×2 plain image *P* is firstly converted into a bit stream B1 and the bits are grouped into 16 2-bit units. We get an index vector idx from the first 16 elements of the given hyperchaotic sequence *H*. It can be further grouped into two small vectors: I1 and I2. Then, we use I1 to rearrange the 16 units to get the permutated PB1. Since the RB is equal to 0 for this 2-bit permutation, there are no remaining bits needed to be embedded. Up to now, the 2-bit permutation completes. The obtained PB1 by the 2-bit permutation is actually the cipher image C1, which is clearly different from *P*. Then, it starts to conduct 3-bit permutation on the PB1. The 32 bits can be grouped into 10 complete 3-bit units and 2 remaining bits, as shown by B2. For the 10 3-bit units, we can rearrange them by I2, and then obtain the PB2 before embedding. $H_{27}$ can be mapped to an integer 5 using Equation (6), and the remaining 2 bits can be inserted after the 5-th 3-bit unit in PB2, shown as PB3 after embedding in the figure. The final PB3 is actually the cipher image C2, a totally different image from *P*. From this illustration, we can see that the proposed *n*-bit permutation can also cause the change of the pixel values in plain images.

### 3.3. Multiple Bit Diffusion

The purpose of diffusion is to change the values of image data. The existing image encryption schemes mainly conduct diffusion on pixel-level data and/or DNA-level data (two bits). Similar to *n*-bit permutation, we propose *n*-bit diffusion that can be conducted on *n*-bit data per unit.

With the *B*, *L*, *H*, and *n* given for *n*-bit permutation, the number of *n*-bit units to be diffused DU is equal to ⌊L/n⌋ and the length of the last unit LL is L%n. If LL equals 0, the last unit is null; otherwise, its length is less than *n* (we call it non-*n*-bit unit) and it needs special handling. In this paper, we use a ciphertext diffusion in crisscross pattern (CDCP)-like idea to conduct *n*-bit diffusion [47]. Specifically, the bit stream *B* is transformed into a vector *P* of *n*-bit unit and then divided into two parts, and the two parts are diffused in crisscross pattern with two rounds. A mask vector *M* and an initial *n*-bit integer *V* can be mapped from *H*. When DU is an even, the first *n*-bit unit of each part can be initialized by Equation (7).

(7) $$\left\{\begin{array}{c} C_{1}=P_{1}⊗\left(\left(V-M_{1}\right)\%2^{n}\right)\\ C_{DU/2+1}=P_{DU/2+1}⊗\left(\left(C_{1}-M_{DU/2+1}\right)\%2^{n}\right) \end{array},\right.$$

where ⊗ is the bitwise XOR (exclusive or) operation, and *C* is the vector of an cipher image. After that, the other *n*-bit units of each part can be updated as Equation (8):

(8) $$\left\{\begin{array}{c} C_{i}=P_{i}⊗\left(\left(C_{DU/2+i-1}-M_{i}\right)\%2^{n}\right)\\ C_{DU/2+i}=P_{DU/2+i}⊗\left(\left(C_{i}-M_{DU/2+i}\right)\%2^{n}\right) \end{array},i=2,3,\cdots ,DU/2.\right.$$

There are two cases that need to be handled specially. When DU is an odd, we use the following formulation to encrypt the (DU+1)/2-th unit.

(9) $$C_{(DU+1)/2}=P_{(DU+1)/2}⊗\left(\left(C_{DU}-M_{(DU+1)/2}\right)\%2^{n}\right).$$

Another case is about the non-*n*-bit unit. When it exists, we use the following formulation which is similar to Equation (9) to handle it.

(10) $$C_{DU+1}=P_{DU+1}⊗\left(\left(C_{DU}-M_{DU+1}\right)\%2^{LL}\right).$$

The second round diffusion is the same as the first round, except that $C_{DU}$ is used as the initial value to replace *V* in Equation (7).

The *n*-bit diffusion can be described as Algorithm 2.
**Algorithm 2***n*-bit diffusion.**Input:** 
a bit stream *B*, a hyperchaotic sequence *H*, and the number of bits to be diffused in a unit *n***Output:** 
a diffused bit stream DB, the number of used elements PU in *H*1:**function**BitDiffuse(*B*, *H*, *n*)2:    *L*← length(*B*); //length of *B*3:    DU←⌊L/n⌋;4:    LL←L%n;5:    P←reshape(B(1:DU∗n),[DU,n]); //reshape the first DU∗n bits in *B* into a vector *P* having DU*n*-bit units6:    $P_{DU+1}=B\left(DU*n+1:end\right)$ //Use $P_{DU+1}$ to denote the remaining L%n bits in *B* if they exist;7:    PU←L/8;8:    Map H(1:PU) to a vector of 8-bit unsigned integers *M*;9:    Map H(PU+1) to a *n*-bit unsigned integer *V*;10:    PU←PU+1;11:    Conduct the first round diffusion with *P*, *V* and *M* by Equations (7)–(10);12:    P←C, $V\leftarrow C_{DU}$;13:    Conduct the second round diffusion with *P*, *V* and *M* by Equations (7)–(10);14:    DB=reshape(P,[1,L]);15:    **return** DB, PU;16:**end function**

**Figure 4:**
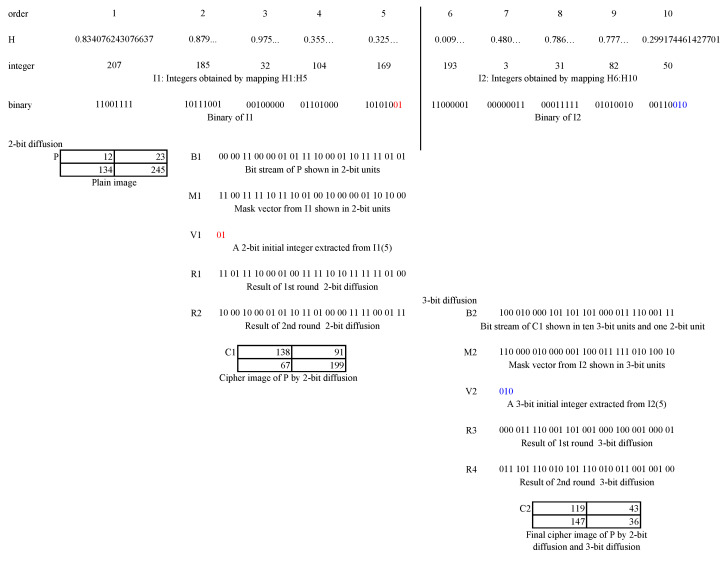
Illustration of 2-bit and 3-bit diffusion.

An illustration on 2-bit diffusion and 3-bit diffusion is shown in Figure 4.

As done in Figure 3, the same plain image *P* is transformed into a binary sequence B1. A hyperchaotic sequence *H* having 10 elements are mapped into 8-bit integers and a further binary sequence (“binary” in the figure) by Equation (6). The sequence can also be split into I1 and I2. Note that some elements in *H* only show their first four digits to save spaces of the figure. The I1 can be further shown in a two-bit format as M1. The initial values V1 is extracted from the last 2 bits from I1(5), as shown in red. When the 2-bit diffusion completes using Equation (7)/Equation (8) for the first/second round, we can obtain R1 and R2, respectively. R2 is actually the cipher image C1, which is totally different from the plain image *P*. Similarly, C1 can be encrypted by performing 3-bit diffusion. Note that since it has a 2-bit unit, when all the 3-bit units are encrypted, the remaining 2-bit unit needs to be encrypted by Equation (10). After the first and the second round 3-bit diffusion, we can obtain R3 and R4, respectively. R4 represents the final cipher image C2, where we can not find any visually information of the plain image *P*.

### 3.4. MBPD: Multiple Bit Permutation and Diffusion for Image Encryption

The main characteristic of the proposed MBPD lies in the permutation and diffusion can be conducted on multiple bit level data, which is very different from the common 1-bit, 2-bit (DNA) and/or 8-bit (pixel) permutation and 8-bit diffusion operations in most existing image encryption schemes.

With the aforementioned analysis, the detailed steps of the proposed MBPD are described as Algorithm 3.
**Algorithm 3** MBPD: Multiple bit permutation and diffusion.**Input:** 
a plain image *P*, initial values $IV=(x_{0},y_{0},z_{0},w_{0})$ for the hyperchaotic system, and iteration numbers of the discarded sequence $it_{0}$**Output:** 
a cipher image *C*1:**function**MBPD(*P*, IV,$it_{0}$)2:    Generate a hyperchaotic sequence *H* with IV and $it_{0}$ as described in Section 3.1;3:    Get the height *h* and the width *w* of *P*;4:    Convert *P* to a bit stream *B* of length L=h∗w∗8;5:    i←0;6:    **for** n=1→8 **do**7:        B,ul←BITPERMUTE(B,H(i+1:end),n);    //*n*-bit permutation8:        i←i+ul;9:        B,ul←BITDIFFUSE(B,H(i+1:end),n);    //*n*-bit diffusion10:        i←i+ul;11:    **end for**12:    Convert *B* to an image *C*;13:    **return** *C*;14:**end function**

The key steps of Algorithm 3 consist of a hyperchaotic sequence generation (Line 2), conversion the plain image to a bit stream (Line 3–4), conducting multiple bit permutation and diffusion on the bit stream (Line 6–11), and converting the bit stream back to an image (Line 12). Note that Algorithm 3 is proposed for gray images, but it can be easily extended for color images. The easiest way is to consider an RGB color image as three gray images and encrypt each gray image independently. The current proposed algorithm considers 8-bit permutation and diffusion at most, and it might be extended for 9-bit, 10-bit and even more bit permutation and diffusion. In addition, the proposed MBPD can be performed more than one round to enhance the effect of encryption. On the other hand, in real-world applications, it is not necessary to conduct all *n*-bit (n=1,2,⋯,8) operations to save time. The proposed MBPD can also be considered as a typical application of the strategy of “divide and conquer” [48,49].

To obtain a decrypted image, it only needs to execute the steps in Algorithm 3 reversely.

## 4. Experimental Results

### 4.1. Experimental Settings

We select the initial values $IV=(x_{0},y_{0},z_{0},w_{0})$ for the presented 4D hyperchaotic system as the security keys of the MBPD. Instead of conducting all bit levels permutation and diffusion, we only perform 6 types of *n*-bit permutation (n=1,2,3,5,6,7) and 2 types of *n*-bit diffusion (n=4,8). Specifically, we list all the parameters in Table 1. Although we use fixed security keys for all test images, they can also be optimized by evolutionary algorithms for each image [50,51,52].

We use 16 publicly accessible 256-level gray images as test images in most experiments. The size of each image is 256×256 or 512×512. We name each image by the format of “name+width”. For example, “Lena512” represents gray Lena image of size 512×512. To demonstrate the performance of the proposed MBPD, we compare it with three popular gray image encryption schemes in most experiments: DFDLC [13], HCDNA [38], and CDCP [47].

All the experiments are conducted with MATLAB R2020b on a PC with 64-bit Windows 10 OS, an i5-9500 CPU @3.00 GHz, and 32 GB RAM.

### 4.2. Security Key Analysis

The security key is very important in cryptography, regardless of whether the encryption object is text, ordinary data or multimedia information. Key space and key sensitivity are two important indicators for evaluating security keys in image encryption.

#### 4.2.1. Key Space

A good encryption scheme should have an enough large key space. An image encryption scheme with a key space larger than $2^{100}$ is able to resist brute-force attacks from modern computers. As far as the proposed MBPD is concerned, the initial value of the 4D hyperchaotic system can be considered as the security key. According to the IEEE standard, the precision of each element of the initial values is $10^{-15}$; hence, the total key space is ${\left(10^{15}\right)}^{4}\approx 2^{199}$, which is far larger than $2^{100}$. In addition, the parameters of the hyperchaotic system, the iteration number to generate discarded sequence, and the combination of permutation and/or diffusion at *n* bits can be thought of as parts of security key to further enlarge the key space. Therefore, the key space of the proposed MBPD is so large that it can resist brute-force attacks.

#### 4.2.2. Key Sensitivity

**Figure 5:**
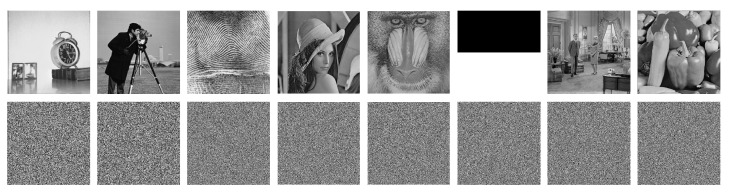
Results of key sensitivity. The first row and the second row show the decrypted images by $K_{1}$ and $K_{2}$, respectively. From left to right: Clock256, Cameraman256, Finger512, Lena512, Baboon512, Bw512, Couple512, and Peppers512.

A hyperchaotic system is extremely sensitive to the key. A tiny change in the key will produce a different hyperchaotic sequence and, hence, result in completely different decrypted images. To demonstrate the sensitivity of the proposed MBPD, we use the corrected security $K_{1}=\left(x_{0},y_{0},z_{0},w_{0}\right)=\left(0.12,0.23,0.34,0.45\right)$ and a slightly different key $K_{2}=\left(x_{0}+10^{-15},y_{0},z_{0},w_{0}\right)$ to decrypt some cipher images. The decrypted images with $K_{1}$ and $K_{2}$ are shown in the first row and the second row of Figure 5, respectively.

From this figure, we can find that the MBPD can decrypt the cipher images correctly with $K_{1}$ and even a tiny change ($10^{-15}$) that occurs in one element of $K_{1}$ will result in random-like images. It reveals that the MBPD is very sensitive to the security key.

To quantitatively demonstrate the sensitivity, we further use the SSIM to measure the structural similarity between the two decrypted images with $K_{1}$ and $K_{2}$ [53]. The lower the SSIM value, the higher the sensitivity. If the SSIM value is very close to 0, it reveals that the two images are almost completely different. Therefore, if a tiny change in the security key produces an SSIM value close to 0, we can say that the security key is very sensitive, from the review of decrypted images. The SSIM values of the decrypted images in Figure 5 are listed in Table 2. From this table, we can observe that all the SSIM values are very close to 0, showing the sensitivity of security keys.

We also use the SSIM to verify the structural similarity between the two cipher images by $K_{1}$ and $K_{2}$. The results are shown in Table 3. Again, we can find that the SSIM values are far below 0.01 and very close to 0, indicating that the security keys are very sensitive to cipher images.

In summary, both the visual decrypted images and the quantitative analysis for decrypted images and cipher images show that the proposed MBPD has sensitive security keys for image encryption.

### 4.3. Statistical Analysis

In this subsection, we will analyze the MBPD via information entropies, histograms and correlations, which are all among the typical statistical analysis indicators in the area of image encryption.

#### 4.3.1. Information Entropy

Entropy is an important concept in physics, communication, information theory, and others. It is often used to measure the uncertainty or randomness of a specific complex system. Given an *L*-level gray image *I* and the probability $p_{i}$ of each gray level *i* occurs in the image, the information entropy of *I*, denoted by E(I), can be calculated by:

(11) $$E\left(I\right)=-\sum_{i=0}^{L-1}p_{i}log_{2}\left(p_{i}\right).$$

For a 256-level test image in the experiment, if it has only one level, for example, all-white image, its information entropy will equal the minimal value, 0. If each level appears with an identical probability, $\frac{1}{256}$, the corresponding information entropy is equal to the maximal value, 8. Therefore, the closer the information entropy to 8, the better the encrypted image. We list the information entropies of all plain images and their corresponding cipher images by the MBPD and the other compared schemes in Table 4, where the highest entropy of each image is shown in bold.

We can see that all plain images’ information entropies are much less than those of their cipher images. Specifically, the entropies of plain images fall in the range of [1.0000,7.6548], where the lower bound and the upper bound are achieved by Bw512 and House512, respectively. However, those values of cipher images of size 256×256 are greater than 7.9957. For cipher images of size 512×512, except for HCDNA’s 7.9154 for Bw512, the lowest information entropy is 7.9920, which is very close to the maximal value, 8. The proposed MBPD, DFDLC, HCDNA, and CDCP achieve the highest information entropies in 8, 10, 1, and 6 out of 16 cases, respectively. In terms of information entropy, the MBPD significantly outperforms HCDNA and achieves comparable results with CDCP and DFDLC, revealing that the MBPD is able to resist entropy attacks effectively.

#### 4.3.2. Histogram

**Figure 6:**
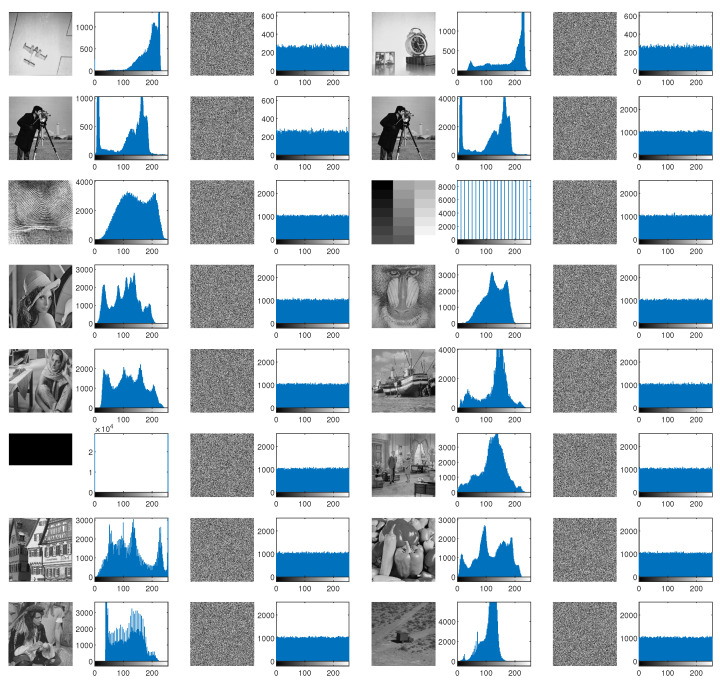
Histograms of plain images and their corresponding cipher images. Each plain image is followed by its histogram, the corresponding cipher image, and its histogram.

The histogram of an image reflects the distribution of pixel levels. A natural image often has a histogram with certain irregular shapes, such as mountain peaks and valleys. A well-designed encryption scheme should break the original distribution of gray-levels and make the new distribution as even as possible. The histograms obtained by the MBPD are shown in Figure 6, where the test images’ orders are the same as in Table 4.

From this figure, we can find that all the natural images (those except for Gray512 Bw512) appear irregular histograms. Since plain Gray512 and Bw512 have evenly 21 and 2 gray-levels, respectively, they only have 21 and 2 bars in the histograms. However, the distributions of the pixel values of the cipher images are so uniform that the tops of the bars in the histograms appear as horizontal lines, even for Gray512 and Bw512. The results reveal that the proposed MBPD can effectively break the distributions of cipher images and produce sufficiently uniform histograms.

#### 4.3.3. Correlation

Strong correlation among neighboring pixels is a key attribute of plain images. A practical image encryption scheme should reduce such correlation significantly. The lower the correlation in cipher images, the better an encryption scheme. Given two sequences $s_{1}$ and $s_{2}$, the correlation (γ) between them can be computed by:

(12) $$\gamma =\frac{\rho (s_{1},s_{2})}{\sqrt{D\left(s_{1}\right)D\left(s_{2}\right)}},$$

where ρ denotes the covariance of two sequences, and *D* is the standard deviation of a sequence. According to this equation, the highest value of correlation will be 1 if $s_{1}$ and $s_{2}$ are identical, while it will be 0 if they are independent.

Given an image, there are many ways to construct $s_{1}$ and $s_{2}$. Typically, when a pixel is put into $s_{1}$, its horizontal, vertical, or diagonal adjacent pixel can be placed in $s_{2}$. In this way, we can use Equation (12) to calculate the correlations at the horizontal ($\gamma_{h}$), vertical ($\gamma_{v}$), and diagonal ($\gamma_{d}$) directions. We use all the pixels in an image to construct $s_{1}$, and then construct corresponding $s_{2}$ to compute $\gamma_{h}$, $\gamma_{v}$, and $\gamma_{d}$. The correlations of plain images and cipher images are shown in Table 5, where the best results are in bold.

From this table, we can observe that all the plain images have high correlations. In particular, the $\gamma_{h}$ of plain Bw512 is equal to the highest value, i.e., 1. However, these high correlations are reduced to a very low level by the encryption schemes. More specifically, the correlations by the encryption schemes are very close to or even equal to 0, showing that all the schemes can break the high correlations in plain images. As far as the four schemes, MBPD achieves the lowest correlations in 15 out of 48 times, followed by CDCP’s 13 times, DFDLC’s 12 times, and HCDNA’s 11 times, indicating that MBPD performs better than the compared encryption schemes.

**Figure 7:**
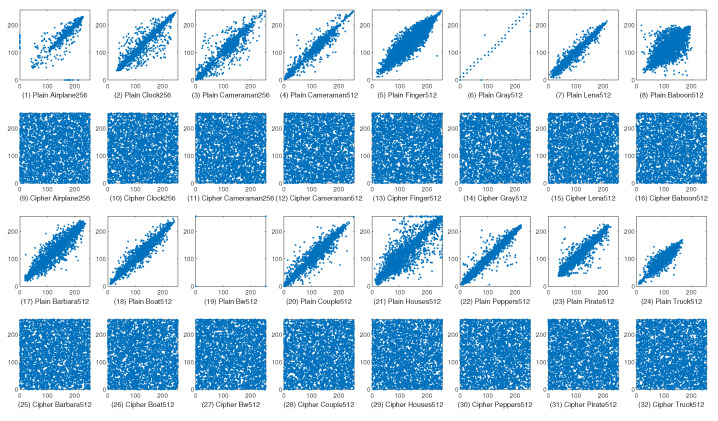
Horizontal correlations of plain images and their corresponding cipher images.

To further analyze the correlations, we randomly pick up 4000 pairs of horizontally adjacent pixels from plain images and cipher images by the proposed MBPD and then plot their gray levels as *x*-values and *y*-values in a 2D plane, as shown in Figure 7. We can observe that the plots of all the plain images except for Bw512 appear near the main diagonals, showing that there exist strong correlations in the cipher images. Since Bw512 has only two gray levels: 0 and 255, most points are piled up at (0,0) and (255,255), which are also on the main diagonal. In contrast, the plots of all the cipher images fill with the whole planes, suggesting low correlations in cipher images.

### 4.4. Differential Attack Analysis

Differential attacks compare the variations in a plain image with variations in the cipher image to find the plain image and/or desired security key. To resist differential attacks, a well-designed image encryption scheme must produce a completely different cipher image even for a tiny change in the corresponding plain image.

There are two popular indicators in the community of image security to measure image encryption schemes’ capability of resisting differential attacks. One is the number of pixels change rate (NPCR), which can be defined as Equation (13). And the other is the unified average changing intensity (UACI) defined by Equation (14).

(13) $$NPCR=\frac{\sum_{h=1}^{H}\sum_{w=1}^{W}d\left(h,w\right)}{H\cdot W}\times 100\%,$$

(14) $$UACI=\frac{\sum_{h=1}^{H}\sum_{w=1}^{W}\left|C_{1}\left(h,w\right)-C_{2}\left(h,w\right)\right|}{255\cdot H\cdot W}\times 100\%,$$

where *H* and *W* denote the height and the width of the cipher images $C_{1}$ and $C_{2}$, and d(h,w) is used to judge whether the gray levels of $C_{1}$ and $C_{2}$ at the position (h,w) are different, as formulated by Equation (15).

(15) $$d\left(w,h\right)=\left\{\begin{array}{cc} 0, & C_{1}\left(h,w\right)=C_{2}\left(h,w\right)\\ 1, & C_{1}\left(h,w\right)\neq C_{2}\left(h,w\right) \end{array}.\right.$$

Given two 8-bit gray images, if they are identical, their both NPCR and UACI obtain the minimal value, 0. If one is all-white and the other is all-black, their NPCR and UACI values will be the maximal value, 1. Since the cipher images are all random-like, the NPCR and UACI values of a pair of cipher images usually fall into a certain range. The study by Wu et al. reveals that, given a significance level α=0.05 and a 256×256 8-bit gray levels image, if the NPCR is greater than $N_{0.05}^{1}=99.5693\%$ and the UACI falls into the range of $\left(U_{0.05}^{11},U_{0.05}^{1u}\right)=\left(33.2824\%,33.6447\%\right)$, the encryption scheme is said to pass NPCR test and UACI test separately at α=0.05 [54]. Similarly, for a 512×512 image, the corresponding NPCR threshold and UACI range are $N_{0.05}^{2}=99.5893\%$ and $\left(U_{0.05}^{21},U_{0.05}^{2u}\right)=\left(33.3703\%,33.5541\%\right)$, respectively.

We compute NPCR and UACI values from the cipher image by the exact plain image and a cipher image by a slightly changed plain image generated by adding one to the least significant bit of a random pixel. The computation procedure is repeated 20 times, and the average NPCR and UACI are reported in Table 6 and Table 7, respectively, where the values that pass the tests are shown in bold. Moreover, the times of passing the test, the standard deviation, and the average value of the 16 test images by each scheme are shown in the last three lines of the tables.

From Table 6, we can find that the MBPD passes the NPCR test on all images, following by DFDLC and CDCP’s in 15 out of 16 cases. The HCDNA fails to the test because it has no operations to expand a tiny change in the plain images to the whole cipher images. Although CDCP achieves the highest average NPCR value (99.6773%) for the 16 test images, but its standard deviation (0.0723%) is not as low as that of MBPD (0.0037%), indicating that the MBPD achieves the stablest NPCR values. Regarding UACI, again, MBPD passes the test on all test images and achieved the lowest standard deviation, and CDCP and DFDLC fails one image, i.e., Bw512 and Pirate512, respectively. HCDNA performs the worst and fails all the test images. To summarize, the proposed MBPD outperforms the other compared schemes in terms of NPCR and UACI and can effectively resist differential attacks.

### 4.5. Robustness

From the above analysis, we know that a tiny change in a plain image will result in a completely different cipher image for a well-designed image encryption scheme. However, contamination in cipher images is unavoidable during transmission and storage. Therefore, a good encryption scheme should recover a contaminated cipher image to some extent. Noise and cropping are two typical types of contamination.

**Figure 8:**
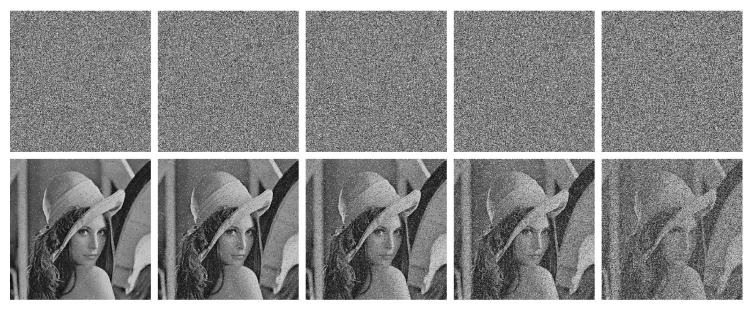
Noise test. The first row, from left to right: cipher images with 0.5%, 1%, 2%, 4%, and 10% salt-and-pepper noise added. The second row: the decrypted images from the corresponding cipher images in the first row.

**Figure 9:**
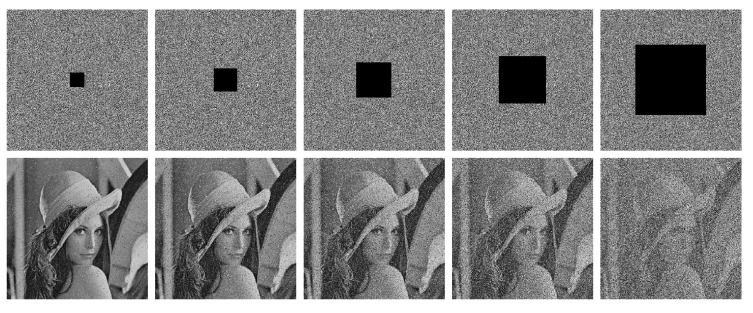
Cropping test. The first row, from left to right: cipher images with 1%, 2.78%, 6.75%, 11.11%, and 25% data loss. The second row: the decrypted images from the corresponding cipher images in the first row.

To validate the robustness to noise and cropping, we first add 0.5%, 1%, 2%, 4%, and 10% salt-and-pepper noise to the cipher images, and decrypt them with the proposed MBPD. The results are shown in Figure 8, where we can find that when the noise level is less than 4%, the MBPD can recover the cipher images very well and even for 10% noise level, the profile of Lena can be clearly recognized. Then, we crop the images with 1%, 2.78%, 6.75%, 11.11%, and 25% data loss, the cropped cipher images and the corresponding decrypted images are shown in Figure 9. We can see that Lena can be easily recognized when the data loss levels are less than 11.11%. When the level equals to 25%, it is hard to recognize the profile of Lena. Another finding is that, even if the data loss is concentrated in the center of an encrypted image, the contaminated locations in the decrypted image are evenly distributed throughout the image.

To summarize, the MBPD can effectively resist noise and cropping attacks to some extent.

### 4.6. Running Time

Running time is used to measure the efficiency of the encrypted algorithms. Table 8 lists the running time of encryption and decryption operations on images with sizes 256×256 and 512×512. We can find that CDCP takes the least time among the four schemes, while the HCDNA takes the most time. The running time of HCDNA is about 30 times that of CDCP. The results of MBPD and DFDLC are somewhere in between and are very close but the former is slightly less than the latter. The major reasons why MBPD is somewhat time-consuming are that it conducts encryption at multiple bit levels and the operations with most multi-bit levels involve string operations. Two possible directions for decreasing running time are: using parallel computing and reducing the number of bit levels for multi-bit operations, e.g., encrypting images only with 1-bit permutation and 4-bit diffusion.

### 4.7. Discussion

From the above experimental results and the corresponding analysis, we can see that the proposed MBPD is a promising scheme for image encryption.

In addition to the proposed 4D hyperchaotic system and the extensive experiments, the major contribution of the paper lies in proposing a novel multiple bit permutation and diffusion scheme for image encryption. The MBPD can encrypt images not only with 1-bit, 2-bit, and 8-bit (one pixel) data that are widely processed by existing image encryption schemes but also with 3–7 bit data that few studies have focused on.

The proposed MBPD’s main advantage over the existing image encryption schemes is that it can perform permutation and diffusion with multiple different bits. The diversity of each encrypted unit’s length is enhanced, and the proposed MBPD finally achieves promising results in terms of the evaluation metrics when compared with four state-of-the-art image encryption schemes, as demonstrated by the experiments.

Sixteen publicly accessible 256-level gray images of two sizes are used to evaluate the proposed MBPD. They include 14 natural images in different scenes, as well as two handcrafted images, which are very popular in the evaluation of image encryption schemes. The MBPD performs quite well with all the test images. Although the MBPD is proposed to encrypt gray images only in this paper, it can be easily extended for color image encryption. The simplest way is to treat each channel of a color image as a gray image, and each channel can be separately encrypted by the MBPD. Here, we use miscellaneous images of different sizes, different scenes and different channels (a 3-channel image means a color image) from the SIPI image database (http://sipi.usc.edu/database/database.php?volume=misc, accessed on 19 April 2021) to verify the generality of the proposed MBPD. Note that the data set has 39 images in total, consisting of 24 gray images and 15 color ones. Six of them have been tested in the above experiments; hence, they are excluded in this experiment. The results of entropy, $\gamma_{h}$, $\gamma_{v}$, $\gamma_{d}$, NPCR, and UACI of the rest 33 images obtained by the proposed MBPD are reported in Table 9, where the test images are sorted by size and image name. Note that the table reports the average of the three channels for color images.

From this table, we can find that the experimental results are very ideal in terms of all the evaluation indicators, regardless of the image content, size, and the number of channels. Specifically, the entropies are very close to the theoretical best value, 8, and all the correlations in all directions are close to 0. All the images pass the NPCR and UACI tests. Therefore, the extensive test images demonstrate that the proposed MBPD has good generality.

## 5. Conclusions

Most existing image encryption schemes involve 1-bit level, 2-bit level (DNA computing), and/or 8-bit level (pixel) data. Few studies focus on other bit-level data, which limits the diversity of encrypted data units and ultimately negatively affects the encryption effect. To this end, this paper proposes a novel multi-bit permutation and diffusion scheme (MBPD) for image encryption. The key characteristic of MBPD is that it can perform permutation and diffusion at different bit-level data, such as 1-bit permutation, 3-bit diffusion, and 6-bit permutation, to encrypt images. The results of extensive experiments demonstrate that the proposed MBPD can resist different types of attacks and has high security. One limitation of the MBPD is that it is somewhat time-consuming. In the future, we will study how to speed it up and apply it to color image encryption.

## Figures and Tables

**Table 1:** Experiment parameters.

Parameter Description	Value
Hyperchaotic system’s parameters	(a,b,c,d,e,f)=(35,7,35,5,1.5,1)
Security keys	$\left(x_{0},y_{0},z_{0},w_{0}\right)=\left(0.12,0.23,0.34,0.45\right)$
Iteration number to generate discarded sequence	$it_{0}=500$
Bit levels of permutation	n=1,2,3,5,6,7
Bit levels of diffusion	n=4,8
Rounds of encryption	1

**Table 2:** The SSIM values of decrypted images with $K_{1}$ and $K_{2}$.

Image Name	SSIM Value	Image Name	SSIM Value
Clock256	0.0083	Cameraman256	0.0087
Finger512	0.0081	Lena512	0.0093
Baboon512	0.0107	Bw512	0.0047
Couple512	0.0110	Peppers512	0.0098

**Table 3:** The SSIM values of cipher images with $K_{1}$ and $K_{2}$.

Image Name	SSIM Value	Image Name	SSIM Value
Clock256	0.0011	Cameraman256	0.0021
Finger512	0.0052	Lena512	0.0081
Baboon512	0.0087	Bw512	0.0060
Couple512	0.0052	Peppers512	0.0054

**Table 4:** Information entropies of the testing images.

Image Name	Plain Image	Cipher Image			
		MBPD	DFDLC [13]	HCDNA [38]	CDCP [47]
Airplane256	6.4523	7.9970	7.9974	7.9961	**7.9975**
Clock256	6.7057	7.9970	**7.9974**	7.9957	7.9972
Cameraman256	7.0492	**7.9976**	7.9972	7.9961	7.9975
Cameraman512	7.0480	**7.9994**	7.9992	7.9982	7.9993
Finger512	6.7279	**7.9994**	7.9993	7.9991	7.9993
Gray512	4.3923	7.9992	**7.9993**	7.9920	**7.9993**
Lena512	7.4460	7.9993	**7.9994**	7.9989	7.9993
Baboon512	7.1391	**7.9994**	**7.9994**	7.9993	7.9993
Barbara512	7.6321	7.9993	**7.9994**	7.9993	7.9993
Boat512	7.1914	7.9992	**7.9994**	7.9990	7.9993
Bw512	1.0000	7.9992	7.9992	7.9154	**7.9993**
Couple512	7.0572	7.9993	7.9993	7.9992	**7.9994**
Houses512	7.6548	**7.9993**	**7.9993**	**7.9993**	7.9992
Peppers512	7.5925	**7.9994**	**7.9994**	7.9992	**7.9994**
Pirate512	7.2367	**7.9993**	**7.9993**	7.9990	**7.9993**
Truck512	6.0274	**7.9994**	**7.9994**	7.9991	7.9993

**Table 5:** The correlation coefficients γ of the testing images.

Image Name	γ	Plain Image	Cipher Image			
			MBPD	DFDLC [13]	HCDNA [38]	CDCP [47]
Airplane256	$\gamma_{h}$	0.9562	−0.0062	0.0004	−0.0049	**−0.0003**
	$\gamma_{v}$	0.8742	**0.0006**	−0.0042	−0.0045	**−0.0006**
	$\gamma_{d}$	0.8995	0.0019	**0.0001**	0.0038	−0.0022
Clock256	$\gamma_{h}$	0.9540	−0.0024	0.0034	**0.0017**	0.0020
	$\gamma_{v}$	0.9734	−0.0107	0.0022	−0.0060	**−0.0017**
	$\gamma_{d}$	0.9376	**0.0013**	0.0026	−0.0015	−0.0031
Cameraman256	$\gamma_{h}$	0.9554	−0.0059	0.0015	**−0.0006**	−0.0013
	$\gamma_{v}$	0.9710	0.0007	0.0023	−0.0012	**0.0001**
	$\gamma_{d}$	0.9377	0.0052	−0.0053	**0.0012**	−0.0030
Cameraman512	$\gamma_{h}$	0.9830	−0.0013	−0.0016	−0.0014	**0.0011**
	$\gamma_{v}$	0.9887	**0.0014**	0.0015	0.0029	−0.0026
	$\gamma_{d}$	0.9746	−0.0017	**0.0002**	−0.0022	0.0010
Finger512	$\gamma_{h}$	0.9343	0.0022	−0.0010	−0.0012	**−0.0002**
	$\gamma_{v}$	0.9168	0.0007	−0.0012	**−0.0005**	**−0.0005**
	$\gamma_{d}$	0.8664	0.0017	**0.0001**	−0.0003	−0.0003
Gray512	$\gamma_{h}$	0.9913	0.0028	−0.0006	0.0017	**0.0001**
	$\gamma_{v}$	0.9989	**0.0009**	0.0021	−0.0010	0.0017
	$\gamma_{d}$	0.9964	**0.0005**	−0.0006	−0.0007	0.0007
Lena512	$\gamma_{h}$	0.9705	**0.0005**	0.0014	0.0022	−0.0028
	$\gamma_{v}$	0.9856	**0.0002**	−0.0004	−0.0004	0.0038
	$\gamma_{d}$	0.9649	**0.0000**	0.0021	−0.0008	0.0023
Baboon512	$\gamma_{h}$	0.8652	**−0.0018**	0.0024	0.0020	−0.0024
	$\gamma_{v}$	0.7524	−0.0017	**−0.0000**	0.0027	0.0024
	$\gamma_{d}$	0.7210	0.0022	0.0026	**0.0011**	0.0012
Barbara512	$\gamma_{h}$	0.8940	**0.0000**	0.0001	0.0007	−0.0001
	$\gamma_{v}$	0.9572	0.0002	0.0034	−0.0018	**−0.0001**
	$\gamma_{d}$	0.8942	0.0004	−0.0006	**−0.0001**	−0.0014
Boat512	$\gamma_{h}$	0.9368	0.0022	**−0.0015**	−0.0022	−0.0052
	$\gamma_{v}$	0.9709	0.0007	0.0008	**−0.0004**	0.0025
	$\gamma_{d}$	0.9240	**−0.0007**	0.0012	0.0015	0.0021
Bw512	$\gamma_{h}$	1.0000	−0.0013	−0.0009	**−0.0001**	−0.0022
	$\gamma_{v}$	0.9922	0.0031	0.0050	**−0.0016**	−0.0019
	$\gamma_{d}$	0.9961	−0.0011	−0.0019	**0.0002**	−0.0008
Couple512	$\gamma_{h}$	0.9451	0.0013	0.0020	**0.0007**	0.0019
	$\gamma_{v}$	0.9514	0.0011	**0.0002**	0.0020	0.0032
	$\gamma_{d}$	0.9116	−0.0015	−0.0011	−0.0006	**0.0001**
Houses512	$\gamma_{h}$	0.9077	**−0.0013**	0.0028	0.0014	−0.0030
	$\gamma_{v}$	0.9173	−0.0002	**0.0000**	−0.0026	0.0016
	$\gamma_{d}$	0.8439	0.0014	**0.0005**	0.0010	−0.0011
Peppers512	$\gamma_{h}$	0.9733	−0.0009	0.0006	−0.0004	**0.0001**
	$\gamma_{v}$	0.9763	−0.0021	−0.0024	0.0007	**−0.0006**
	$\gamma_{d}$	0.9650	**0.0005**	0.0007	0.0011	−0.0008
Pirate512	$\gamma_{h}$	0.9593	**−0.0006**	0.0014	−0.0020	−0.0020
	$\gamma_{v}$	0.9675	−0.0022	**0.0006**	−0.0008	**0.0006**
	$\gamma_{d}$	0.9432	**0.0002**	−0.0010	−0.0003	−0.0023
Truck512	$\gamma_{h}$	0.9610	0.0005	**0.0000**	0.0012	0.0035
	$\gamma_{v}$	0.9164	0.0018	**0.0001**	0.0003	−0.0004
	$\gamma_{d}$	0.9048	−0.0028	**−0.0003**	−0.0016	−0.0005

**Table 6:** The average NPCR (%) of running the schemes 20 times.

Image	MBPD	DFDLC [13]	HCDNA [38]	CDCP [47]
Airplane256	**99.6014** %	**99.6125**%	76.4828%	**99.6374**%
Clock256	**99.6114**%	**99.6085**%	65.7269%	**99.7081**%
Cameraman256	**99.6099**%	**99.6196**%	73.4785%	**99.7564**%
Cameraman512	**99.6112**%	**99.6078**%	67.1009%	**99.6590**%
Finger512	**99.6083**%	**99.6108**%	76.2949%	**99.6928**%
Gray512	**99.6088**%	**99.6131**%	61.1288%	**99.6767**%
Lena512	**99.6062**%	**99.6084**%	66.5552%	**99.6849**%
Baboon512	**99.6104**%	**99.6077**%	64.3461%	**99.6372**%
Barbara512	**99.6113**%	**99.6114**%	73.5446%	**99.5927**%
Boat512	**99.6093**%	**99.6089**%	75.0493%	99.4786%
Bw512	**99.5997**%	89.6501%	64.8879%	**99.7000**%
Couple512	**99.6033**%	**99.6045**%	63.5847%	**99.7910**%
Houses512	**99.6037**%	**99.6092**%	75.8256%	**99.7578**%
Peppers512	**99.6090**%	**99.6100**%	73.8790%	**99.6849**%
Pirate512	**99.6082**%	**99.6087**%	73.9838%	**99.6765**%
Truck512	**99.6063**%	**99.6070**%	66.5778%	**99.7033**%
Pass/Fail/All	16/0/16	15/1/16	0/16/16	15/1/16
Std.	0.0037%	2.4899%	5.3211%	0.0723%
Mean	99.6074%	98.9874%	69.9029%	99.6773%

**Table 7:** The average UACI (%) of running the schemes 20 times.

Image	MBPD	DFDLC [13]	HCDNA [38]	CDCP [47]
Airplane256	**33.4400** %	**33.4256**%	30.6926%	**33.4682**%
Clock256	**33.4610**%	**33.4992**%	28.3912%	**33.5090**%
Cameraman256	**33.4312**%	**33.4529**%	31.3096%	**33.4766**%
Cameraman512	**33.4618**%	**33.4547**%	27.7148%	**33.4765**%
Finger512	**33.4552**%	**33.4766**%	33.6617%	**33.4796**%
Gray512	**33.4628**%	**33.4638**%	25.1829%	**33.4842**%
Lena512	**33.4545**%	**33.4581**%	27.2038%	**33.4484**%
Baboon512	**33.4590**%	**33.4528**%	26.1169%	**33.4996**%
Barbara512	**33.4905**%	**33.4746**%	28.2405%	**33.5072**%
Boat512	**33.4684**%	**33.4781**%	31.6422%	**33.4881**%
Bw512	**33.4899**%	30.1296%	22.3338%	**33.4655**%
Couple512	**33.4661**%	**33.4853**%	25.9647%	**33.4975**%
Houses512	**33.4682**%	**33.4631**%	31.4138%	**33.4587**%
Peppers512	**33.4409**%	**33.4255**%	29.2497%	**33.4637**%
Pirate512	**33.4808**%	**33.4251**%	30.3032%	33.5917%
Truck512	**33.4612**%	**33.4609**%	28.0393%	**33.4589**%
Pass/Fail/All	16/0/16	15/1/16	0/16/16	15/1/16
Std.	0.0164%	0.8328%	2.8880%	0.0335%
Mean	33.4620%	33.2516%	28.5913%	33.4858%

**Table 8:** Running time of encryption and decryption (in seconds).

Operation	Size	MBPD	DFDLC [13]	HCDNA [38]	CDCP [47]
Encryption	256×256	0.8158	0.8479	3.4635	0.1268
	512×512	3.3833	3.4261	14.2808	0.5240
Decryption	256×256	0.8136	0.8366	4.7181	0.1305
	512×512	3.3624	3.4116	19.5680	0.5126

**Table 9:** Results obtained by the proposed MBPD on miscellaneous images from the SIPI image database.

Image	Size	Entropy	$\gamma_{h}$	$\gamma_{v}$	$\gamma_{d}$	NPCR	UACI
5.1.10	256×256	7.9973	0.0010	−0.0024	−0.0003	99.6053%	33.4448%
5.1.13	256×256	7.9976	0.0011	0.0000	−0.0016	99.6127%	33.4409%
5.1.14	256×256	7.9974	−0.0022	0.0000	−0.0010	99.6114%	33.5188%
Moonsurface256	256×256	7.9973	0.0023	−0.0042	−0.0012	99.6093%	33.4342%
5.2.10	512×512	7.9992	0.0010	−0.0040	0.0005	99.6116%	33.4516%
7.1.02	512×512	7.9993	−0.0007	0.0018	−0.0004	99.6083%	33.4698%
7.1.03	512×512	7.9993	−0.0040	0.0017	−0.0004	99.6070%	33.4621%
7.1.04	512×512	7.9993	0.0001	−0.0006	−0.0027	99.6060%	33.4578%
7.1.05	512×512	7.9993	−0.0017	−0.0027	0.0013	99.6114%	33.4616%
7.1.06	512×512	7.9994	0.0028	0.0015	−0.0027	99.6074%	33.4786%
7.1.07	512×512	7.9993	0.0018	−0.0006	−0.0004	99.6066%	33.4736%
7.1.08	512×512	7.9994	0.0007	−0.0012	−0.0000	99.6093%	33.4505%
7.1.09	512×512	7.9993	0.0003	−0.0035	0.0011	99.6142%	33.4582%
7.1.10	512×512	7.9992	−0.0026	−0.0007	−0.0000	99.6109%	33.4435%
Aerial512	512×512	7.9993	0.0002	−0.0016	0.0004	99.6075%	33.4789%
ruler.512	512×512	7.9993	−0.0045	−0.0001	−0.0010	99.6134%	33.4642%
5.3.01	1024×1024	7.9998	−0.0006	−0.0017	0.0002	99.6117%	33.4545%
5.3.02	1024×1024	7.9998	0.0014	0.0000	0.0004	99.6090%	33.4637%
7.2.01	1024×1024	7.9998	0.0005	−0.0001	−0.0006	99.6127%	33.4609%
4.1.01	256×256×3	7.9969	0.0016	0.0031	0.0027	99.6155%	33.4652%
4.1.02	256×256×3	7.9975	−0.0068	−0.0037	0.0032	99.6149%	33.4376%
4.1.03	256×256×3	7.9971	0.0029	−0.0029	−0.0001	99.6168%	33.4728%
4.1.04	256×256×3	7.9972	0.0013	0.0024	0.0001	99.5991%	33.4596%
4.1.05	256×256×3	7.9974	0.0030	0.0031	−0.0008	99.6046%	33.4304%
4.1.06	256×256×3	7.9971	−0.0030	0.0009	0.0020	99.6051%	33.3983%
4.1.07	256×256×3	7.9972	0.0002	−0.0003	−0.0049	99.6139%	33.4285%
4.1.08	256×256×3	7.9970	0.0024	−0.0008	0.0003	99.6086%	33.4542%
4.2.01	512×512×3	7.9993	0.0014	−0.0005	−0.0006	99.6051%	33.4530%
4.2.03	512×512×3	7.9992	−0.0011	0.0032	0.0017	99.6102%	33.4833%
4.2.05	512×512×3	7.9994	−0.0004	−0.0002	−0.0004	99.6111%	33.4447%
4.2.06	512×512×3	7.9993	0.0004	−0.0016	−0.0006	99.6106%	33.4843%
4.2.07	512×512×3	7.9993	−0.0013	−0.0001	−0.0000	99.6087%	33.4446%
house	512×512×3	7.9992	−0.0012	0.0005	−0.0010	99.6079%	33.4962%

## Data Availability

The used test images are all included in the paper.
